# Enhancement in the antibacterial activity of cephalexin by its delivery through star-shaped poly(ε-caprolactone)-block-poly(ethylene oxide) coated silver nanoparticles

**DOI:** 10.1098/rsos.201097

**Published:** 2020-10-07

**Authors:** Sana Rahim, Samina Perveen, Shakil Ahmed, Muhammad Raza Shah, Muhammad Imran Malik

**Affiliations:** H.E.J. Research Institute of Chemistry, International Centre for Chemical and Biological Sciences (ICCBS), University of Karachi, Karachi 75270, Pakistan

**Keywords:** antibacterial activity, *Staphylococcus aureus*, silver nanoparticles, amphiphilic star block copolymers

## Abstract

The antibacterial activity of silver nanoparticles (AgNPs) stabilized with a four-armed star-shaped poly(ε-caprolactone)-block-poly(ethylene oxide) copolymer [St-P(CL-b-EO)] and its application as a drug delivery vehicle for cephalexin (Cp) was evaluated against pathogenic *Staphylococcus aureus*. The prepared AgNPs were characterized by ultraviolet-visible spectroscopy, Fourier transform infrared spectroscopy, zeta sizer and atomic force microscopy (AFM). The antibacterial efficiency of Cp is enhanced several-fold by its delivery through complexation with St-P(CL-b-EO)-AgNPs, monitored by microplate assay and biofilm destruction studies. Finally, the visual destruction of bacterial cells and its biofilms by employing Cp and its conjugates at their minimum inhibitory concentration (MIC_50_) and minimum biofilm inhibitory concentration (MBIC_50_), respectively, is observed by topographic imaging by AFM. Enhanced antibacterial activity of St-P(CL-b-EO)-AgNPs loaded Cp is attributed to penetrative nature of the drug cargo St-P(CL-b-EO)-AgNPs towards the bacterial cell wall.

## 1. Introduction

Infectious diseases are one of the major reasons of morbidity and mortality across the world [1–3]. According to an estimate, 20–30 per cent of the human population is a longstanding carrier of *Staphylococcus aureus*, a round-shaped gram-positive bacterium that belongs to the phylum Firmicutes. *Staphylococcus aureus* is a natural inhabitant of the normal skin flora in the nostrils and the lower genital part of a women's reproductive system. *Staphylococcus aureus* causes many illnesses, including skin infections such as carbuncles, abscesses, pimples, cellulitis and scalded skin syndrome such as osteomyelitis, sepsis, endocarditis, pneumonia, bacteremia and toxic shock syndrome [4–6]. These pathogenic strains mostly spread infections via the production of potent protein toxins and the cell surface protein expression that inactivates antibodies through binding. In clinical medicines, the development of antibiotic-resistant strains of *S. aureus*, i.e. methicillin-resistant *S. aureus*, is a global problem. Despite extensive research in the field, no immunization is yet approved against *S. aureus* [3,7–9].

Moreover, the infections caused by *S. aureus* are generally treated by cephalexin (Cp) [10,11]. Cp is a first-generation cephalosporin that fits in the class β-lactam antibiotics and exhibits enormous antibacterial activity against both Gram-negative and Gram-positive bacteria [12,13]. However, bacterial communities, especially *S. aureus*, have shown resistance to such traditional antibiotics [11]. Hence, continuous research on the production of active antibacterial agents is extensively required because of the advent of multi-drug-resistant bacterial strains, along with efforts to enhance the efficacy of the existing drugs.

Initial focus of research on nano–biointeractions include studies comprised both the biological compositions as well as material properties, such as analysis of biomolecular signalling, chemical functionality, clearance, transport kinetics, gene expression variations [14], surface charge and toxicity [15]. In this context, the term ‘polymer therapeutics' encompasses polymer-based drugs, polymer protein conjugates, block copolymer micelles, aptamers and multicomponent nonviral vectors with covalent linkages [16]. Polymers allow flexibility and versatility in therapeutic applications owing to the possible modifications based on their functionalization, different synthesis methods, number of variable distributions and diversity [17–19]. However, the application of large-sized materials such as polymers in drug delivery has to encounter numerous challenges, for instance poor solubility, poor bioavailability, *in vivo* instability, poor absorption in the body, issues with target-specific delivery and tonic effectiveness, beside its possible unfavourable effects on the drug efficacy. In this regard, novel drug delivery systems such as polymer-stabilized metal nanoparticles could provide a synergistic combination of properties for targeted drug delivery [20,21]. Furthermore, nanoparticle-based antimicrobial formulation has appeared to act as an effective antibactericidal material. These nanoparticles are sub-colloidal structures (size: 10–100 nm) that serve as efficient drug carriers for enhancement in the drug potency [22,23]. Several authors have presented research articles and reviews signifying the importance, synthesis, characterization and myriad of applications of metal nanoparticles in the modern technological world [24–29].

Silver nanoparticles (AgNPs) with well-known surface chemistry, chemical stability and controlled geometry have been employed for biological applications [30,31]. The enhancement in the antibacterial potential of ampicillin was shown by its combination with gold and silver NPs versus multi-drug-resistant strains including *S. aureus, Enterobacter aerogenes and Pseudomonas aeruginosa [*32]. Naked AgNPs are prone to aggregation, hence surface stabilization of nanoparticles is imperative. The potential of a star-shaped poly(ε-caprolactone)-block-poly(ethylene oxide) copolymer [St-P(CL-b-EO)] as a stabilizing agent for AgNPs is demonstrated in a recent study by our research group [33]. St-P(CL-b-EO), a non-toxic and non-ionic polymer, exhibits excellent properties of biodegradability, biocompatibility and easy excretion from the body. Furthermore, ether and ester groups of St-P(CL-b-EO) contain an oxygen atom that has an affinity to chelate with metal ion which ultimately induces stabilization and prevents aggregation of metal NPs [34–37].

In this study, we report the preparation of AgNPs stabilized with four-armed St-P(CL-b-EO) and studied its proficiency to boost the antibacterial potential of Cp through the determination of minimum inhibitory concentration (MIC) and minimum biofilm inhibitory concentration (MBIC). The overall purpose of the study is to evaluate the potential of complexation of AgNPs with Cp in context of enhancement in its therapeutic efficacy.

## 2. Experimental

### 2.1. Materials and instruments

ε-Caprolactone (ε-CL) and sodium borohydride (NaBH_4_) were purchased from TCI, Japan; methoxy poly(ethylene oxide) (MeO-PEO), maleic anhydride, stannous octoate (Sn(Oct)_2_), silver nitrate (AgNO_3_) and pentaerythritol were purchased from Aldrich, Germany; 4-dimethylaminopyridine was purchased from Across Organics, USA; and trimethylamine by Daijung, Korea were used as received. High performance liquid chromatography grade solvents, chloroform and acetone from RCI Labscan limited, Thailand were used as the reaction medium and for cleaning of apparatus.

Glassware was cleaned with aqua regia to avoid metal contamination, dried in an oven and rinsed with deionized water followed by acetone before use.

A digital pH meter (model 510, Oakton, Eutech) was employed for monitoring the pH of St-P(CL-b-EO)-AgNPs solution. Ultraviolet (UV)-visible spectroscopy was performed on a Shimadzu double beam spectrophotometer, UV-1800 series, operated in the wavelength range of 800–190 nm. A quartz cuvette having path length of 1 cm was used for screening of samples. Bruker Vector 22 Fourier transform infrared spectroscopy (FTIR) spectrometer was used to record FTIR spectra using potassium bromide pellets in the infrared range (400–4000 cm^−1^). Ten scans were used in order to get 0.1 cm^–1^ spectral resolution.

Surface morphology of St-P(CL-b-EO)-AgNps was studied by an Agilent 5500 atomic force microscope, (USA). A drop of St-P(CL-b-EO)-AgNps was dropped on the silicon wafer and dried for 24 h in an inert atmosphere. A zeta sizer (Nano-ZSP, Malvern Instruments) was used to determine particle size distribution and zeta potential of the St-P(CL-b-EO)-AgNps along with their complex with Cp. The study was carried out at a scattering angle of 90° at 25°C. Disposable cuvettes were used to determine zeta size while zeta potential studies were carried out in dip cell cuvettes.

### 2.2. Synthesis of four-armed St-P(CL-b-EO) copolymer

Ring-opening polymerization was employed to synthesize four-armed star-shaped polycaprolactone using pentaerythritol as an initiator and stannous octoate as a catalyst. In parallel, the hydroxyl end-functionality of MeO-PEO is changed to a carboxylic (-COOH) group. Finally, both –OH terminated star-shaped polycaprolactone (PCL) and –COOH terminated MeO-PEO moieties were joined to form a four-armed St-P(CL-b-EO) copolymer. The detailed synthesis protocol and characterization is particularized in our previous publication [38]. The St-P(CL-b-EO) used for this study was a coupled product of star-PCL_10_K and MeO-PEO_2_K_._

### 2.3. Synthesis of St-P(CL-b-EO)-silver nanoparticles

St-P(CL-b-EO)-AgNPs were prepared in a mixture of solvents, composed of acetone and water [33]. The concentration of AgNO_3_, St-P(CL-b-EO) and NaBH_4_ were 1.0, 0.1 and 4.0 mM, respectively. One millilitre of St-P(CL-b-EO) solution was added in 50 ml aqueous AgNO_3_ (an optimized ratio). NaBH_4_ (0.1 ml) was added after 15 min of constant stirring. The colourless reaction mixture turned yellow after 30 min of continuous stirring and indicates the successful synthesis of AgNPs. Detailed characterization of synthesized St-P(CL-b-EO)-AgNPs is particularized in our previous work [33].

### 2.4. Preparation of St-P(CL-b-EO)-silver nanoparticles/cephalexin complex

A 0.1 mM solution of Cp was prepared in distilled water and mixed with equal volume of St-P(CL-b-EO)-AgNPs (1–50) solution to make a St-P(CL-b-EO)-AgNPs/Cp complex. The complex formation was confirmed by UV-visible spectroscopy, atomic force microscopy (AFM), zeta sizer, zeta potential and FTIR as reported in the earlier publication of this series [33].

### 2.5. Determination of loading efficiency

Loading efficiency of the drug in the above-mentioned compelxation is determined through a UV-visible spectrophotometer (Shimadzu, UV-240, Hitachi U-3200). In brief, the above-mentioned formulation of drug and nanoparticles was centrifuged at 14 000 r.p.m. for 25 min. The pellets at the bottom of the Eppendorf tube were carefully collected, followed by the dissolution of these pellets in acetone. In parallel, a calibration curve for the amount of Cp in acetone is constructed by recording UV-visible spectra at 266 nm of solutions of different concentrations of Cp (0.1 to 0.5 mg ml^−1^) which is then employed for the determination of free drug in acetone. The encapsulation efficiency of micelles was calculated by using the following equation:

$$\text{\%}\text{encapsulation}\;\text{efficiency}=\frac{A_{\left(\mathrm{Total}\;\mathrm{drug}\right)}-A_{\left(\mathrm{Free}\;\mathrm{drug}\right)}}{A_{\left(\mathrm{Total}\;\mathrm{drug}\right)}}\times 100,$$

where *A* is the amount of the drug.

### 2.6. Antibacterial assay

#### 2.6.1. Bacterial strains

*Staphylococcus aureus* ATCC 25923, a Gram-positive bacteria, was selected for antibacterial assay. To prepare the stock culture of the bacterial strain, it was kept on tryptic soya agar (Oxford, UK) at 4°C. Initially, the bacterial culture was sub-cultured on a fresh agar plate for 24 h before subjecting it to antibacterial assay. Inocula of *S. aureus* was arranged by vaccinating multiple distinct colonies of bacteria into a liquefied medium of Mueller Hinton broth. The bacterial suspension finally gets homogeneous having a final density of 1 × 10^6^ cfu ml^−1^ that was confirmed by viable counts of colonies.

#### 2.6.2. Microplate assay for growth inhibition efficiency

The bacterial growth inhibition efficiency of the test sample and reference materials, Cp, St-P(CL-b-EO), St-P(CL-b-EO)-AgNPs and St-P(CL-b-EO)-AgNPs/Cp were assessed by 96-well microplate assay. The growth reduction of bacterial cells was quantified through tetrazolium dye reduction methods by a microtiter plate reader (Spectramax) [39]. A concentration of 10^6^ cfu ml^−1^ of freshly collected bacterial cell suspension of *S. aureus* was sown in each well of a 96-well plate. A serial dilution of the above-mentioned materials was conducted in Muller Hinton broth in a range of 10 to 500 µg. In triplicate wells, 200 µl of each concentration was placed and plates were incubated under constant shaking of 150 rpm at 37°C ± 0.5 for 18–24 h. After incubation, 50 µl of 3-(4, 5-dimethylthiazol-2-yl)-2, 5-diphenyltetrazolium bromide (MTT dye) solution (0.2 mg ml^−1^) was added in each well of the 96-well plate, and the plate was again incubated under similar conditions for 30 min. The negative control was taken as dimethyl sulfoxide (DMSO), while positive control was conducted through bacterial suspension. The absorbance was measured at 570 nm by adding DMSO on a spectrophotometer while reference wavelength was 650 nm. The per cent reduction of MTT dye was calculated to indicate the bacterial growth inhibition [40]:

$$\text{growth}\;\text{inhibtion}\;(\text{\%})=\left[\frac{\text{optical density of control}-\text{optical density of test}}{\text{optical density of control}}\right]\;\times 100.$$

#### 2.6.3. Biofilm growth inhibition efficiency

Antibiofilm activity of *S. aureus* was evaluated by using the microtiter biofilm plate method against four selected combinations of Cp, St-P(CL-b-EO), St-P(CL-b-EO)-AgNPs and St-P(CL-b-EO)-AgNPs/Cp. The compounds were diluted in 96-well flat-bottom plates (Corning, USA). An inoculum consisting of 1 × 10^6^ cfu ml^−1^ of bacterial cells were simply inoculated in each well except broth which was taken as a negative control. The plates were incubated at 37°C overnight. To check perfect biofilm formation, the plates were stained with 0.1% (w/v) crystal violet for 20 min after washing thrice with sterile distilled water to remove planktonic cells. The stained plates were rewashed with sterile distilled water and the retained crystal violet-stained biofilms were dissolved in 30% (v/v) glacial acetic acid. The microtiter plate reader (Tecan, USA) was set at 590 nm to take absorbance of plates. Percentage biofilm inhibition was calculated by the following formula:

$$\text{biofilm}\;\text{growth}\;\text{inhibtion}\;(\text{\%})=\left[\frac{\text{optical density of control}-\text{optical density of test}}{\text{optical density of control}}\right]\times 100.$$

#### 2.6.4. Atomic force microscopy

Fresh culture of *S. aureus* was grown up in tryptic soy agar (Oxoid) for 24 h at 37°C. Newly prepared mica slides by cleaving are arranged by adding 10 µl of poly L-lysine which was dried at ambient temperature under the sterilized air flow chamber. A few drops of diluted culture of bacterial strain with the concentration of 10^6^ cfu ml^−1^ were applied on already prepared poly L-lysine mica slides. Five to 10 µl of tested compound samples were also introduced from the respective wells of microtiter plates for inhibition and biofilm inhibition evaluations at their respective MIC_50_ and MIBC_50_. All the slides were dried at ambient temperature under sterile conditions and imaged by AFM for morphological characteristic evaluation of the bacterial cells.

## 3. Results and discussion

### 3.1. Molecular characterization of St-P(CL-b-EO)

As mentioned in the experimental section, St-P(CL-b-EO) was synthesized by coupling of St-PCL_10_K with MeO-PEO_2_K_._ The average number and average weight molar mass of synthesized four-armed St-PCL_10_K as obtained by polystyrene calibrated size exclusion chromatography was 9700 and 13 700 g mol^−1^. MeO-PEO_2_K used in the coupling reaction was a commercial product. The four-armed St-P(CL-b-EO) were comprehensively characterized by size exclusion chromatography, FTIR, ^1^H-NMR and liquid chromatography at critical conditions of both PCL and PEO. Size exclusion chromatography (SEC) elugrams and ^1^H-NMR spectra of precursors MeO-PEO_2_K, and St-PCL_10_K_,_ along with the final product St-P(CL_10 K_-b-EO_2_K) are presented in the electronic supplementary material, figures S1 and S2. The comprehensive characterization of intermediates and final product clearly demonstrate the successful synthesis which is elaborated in great detail in [38].

### 3.2. Synthesis and characterization of St-P(CL-b-EO)-silver nanoparticles

**Figure 1.**
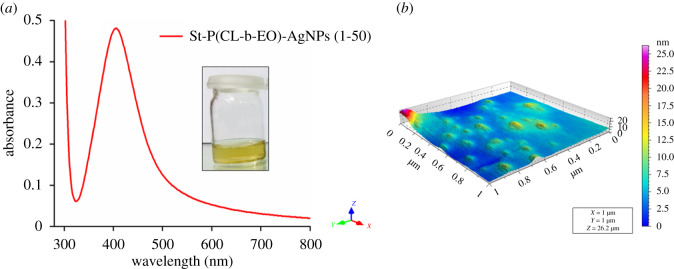
Confirmation of formation of St-P(CL-b-EO)-AgNPs: (*a*) UV-visible spectrum; (*b*) AFM topographic image.

St-P(CL-b-EO) is a four-armed star-shaped block copolymer comprising of PEO as the hydrophilic segment and PCL as the hydrophobic part. St-P(CL-b-EO)-AgNPs were synthesized by NaBH_4_ reduction method of AgNO_3_ in the presence of St-P(CL-b-EO). A characteristic surface plasmon resonance (SPR) band at 415 nm was obtained in the UV-visible spectrum by mixing AgNO_3_ and St-P(CL-b-EO) solutions at an optimized ratio of 50 : 1 (v/v) for 30 min, figure 1*a*. AFM analysis revealed that St-P(CL-b-EO)-AgNps are polydisperse and have size in the range of 5–20 nm figure 1*b*). The inset depicts the pale yellowish colour of stabilized St-P(CL-b-EO)-AgNPs.

**Figure 2.**
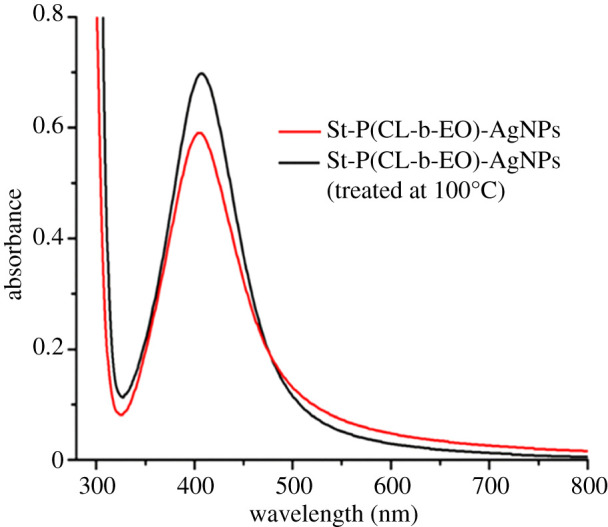
UV-visible spectrum of St-P(CL-b-EO)-AgNPs after incubation at 100°C for 20 min.

The stability of St-P(CL-b-EO)-AgNPs against different external parameters, such as temperature, and the presence of electrolytes and pH are evaluated. Temperature treatment of synthesized St-P(CL-b-EO)-AgNPs at 100°C for 20 min resulted in an increase in the intensity of the SPR band which is an indication of enhanced stabilization (figure 2). Moreover, St-P(CL-b-EO)-AgNPs persisted for more than 12 months at ambient temperature.

**Figure 3.**
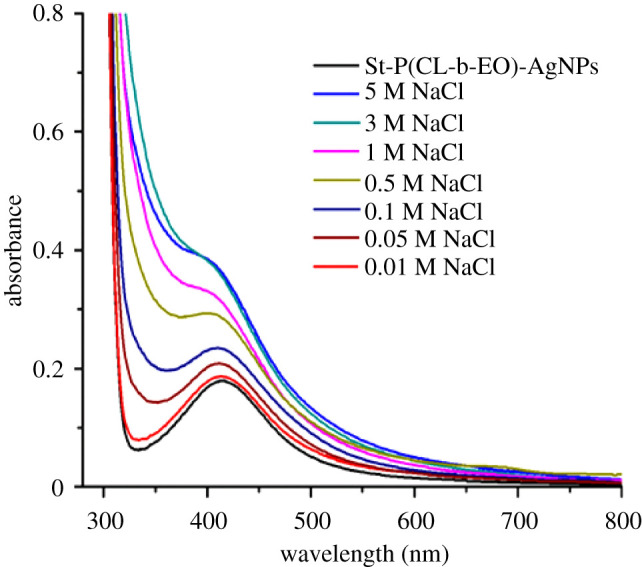
Change in the SPR band of St-P(CL-b-EO)-AgNPs as a function of NaCl concentration (0.01 M-5 M).

To check the stability of St-P(CL-b-EO)-AgNPs in the presence of electrolytes, various electrolyte concentrations (0.01 M–5 M NaCl) were added in St-P(CL-b-EO)*-*AgNPs and their stability is monitored by UV-visible spectroscopy, figure 3. A visible decrease in the sharpness of the absorbance peak is observed by an increase in the electrolyte concentration. Moreover, the typical AgNPs peak just appeared as a shoulder by addition of NaCl beyond 1.0 M. The aggregation of AgNPs by addition of electrolyte is attributed to the presence of a large amount of Cl^−1^ ions in the solution [41,42].

**Figure 4.**
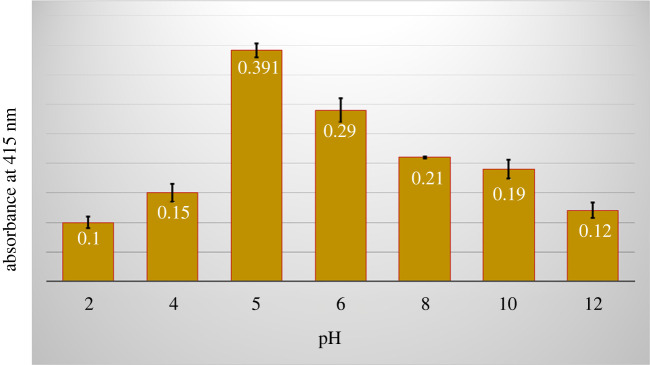
Change in the intensity of SPR band of St-P(CL-b-EO)-AgNPs as a function of pH.

Synthesized St-P(CL-b-EO)*-*AgNPs were slightly acidic in nature having a pH approximately 5. The changes in the pH in a range of 2–12 resulted in a visible reduction in the intensity of the SPR band of St-P(CL-b-EO)*-*AgNPs, figure 4. It is found that synthesized St-P(CL-b-EO)*-*AgNPs have maximum stability at the original pH (approx. 5), as deduced from the intensity of the SPR band. Furthermore, the stability of AgNPs decreased while moving away from original pH, which is more pronounced towards the acidic environment compared to neutral or slightly basic pH.

### 3.3. Complexation of St-P(CL-b-EO)-silver nanoparticles with cephalexin

**Figure 5.**
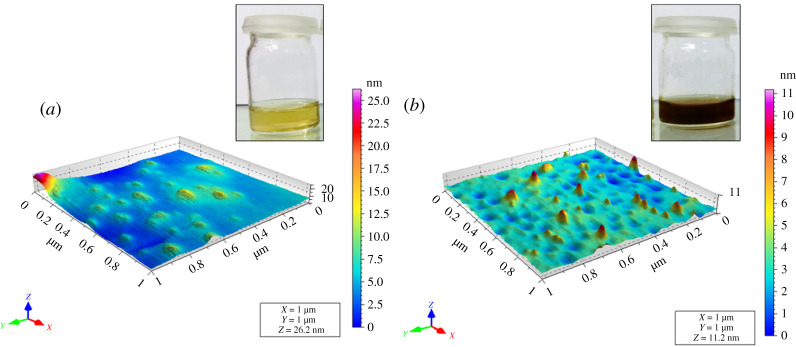
Atomic force micrographs (AFMs), (*a*) P(EO-b-CL)-AgNPs; (*b*) P(EO-b-CL)-AgNPs/Cp.

The addition of Cp in the St-P(CL-b-EO)*-*AgNPs resulted in the formation of a complex that is more stable compared to parent St-P(CL-b-EO)*-*AgNPs. The size of St-P(CL-b-EO)*-*AgNPs/Cp complex decreased (dynamic light scattering and AFM analysis), while stability increased (zeta potential values) compared to St-P(CL-b-EO)*-*AgNPs [33]. The average size of St-P(CL-b-EO)*-*AgNPs decreased from 151.5 ± 8.071 to 140.5 ± 9.895 nm for St-P(CL-b-EO)*-*AgNPs/Cp. Similarly, zeta potential of values of −3.33 ± 4.18 for St-P(CL-b-EO)*-*AgNPs compared to −10.6 ± 4.27 mV for St-P(CL-b-EO)*-*AgNPs/Cp indicates enhanced stability of the latter. Figure 5 demonstrates the AFM images of St-P(CL-b-EO)-AgNPs and St-P(CL-b-EO)-AgNPs/Cp. The typical pale yellow colour of St-P(CL-b-EO)-AgNPs turns dark brown by the addition of Cp in it.

**Figure 6.**
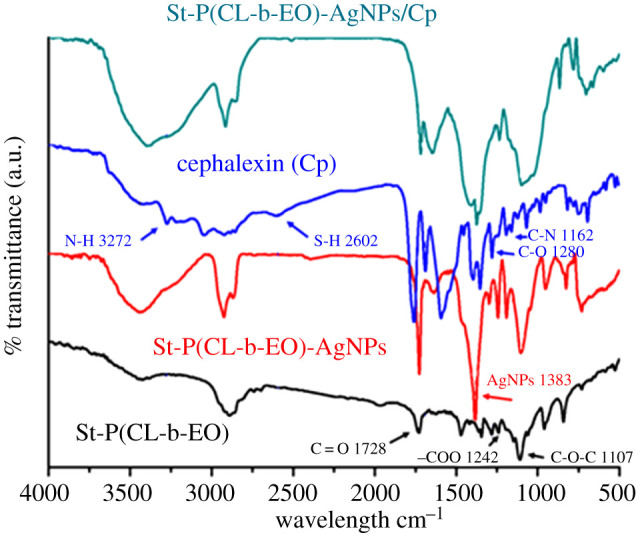
FTIR spectra of St-P(CL-b-EO), Cp, St-P(CL-b-EO)-AgNPs and St-P(CL-b-EO)-AgNPs/Cp complex [33].

Various functionalities present on the Cp, viz. N-H, S-H, C-N, C=O, C-O have a tendency to donate lone pairs of electrons to AgNPs that makes the basis for strong interaction of the drug with the AgNPs. FTIR spectra of St-P(CL-b-EO), St-P(CL-b-EO)-AgNPs and St-P(CL-b-EO)-AgNPs/Cp demonstrate the possible mechanism of NP formation and their complexation with Cp, figure 6. A comparison of the FTIR spectra of St-P(CL-b-EO), and St-P(CL-b-EO)-AgNPs reveal that all the characteristic peaks for the St-P(CL-b-EO) are present after interaction with AgNPs; hence, the non-ionic St-P(CL-b-EO) sterically stabilized the AgNPs. Moreover, the characteristic peak at 1383 cm^−1^ in the spectrum of St-P(CL-b-EO)-AgNPs confirms the formation of AgNPs [42,43]. The FTIR spectrum of Cp has specific peaks at 3272, 2602, 1280 and 1162 cm^−1^ for N-H stretch, S-H stretch, C-O stretch and C-N stretch, respectively. Interestingly, in the FTIR spectrum of St-P(CL-b-EO)-AgNPs/Cp, the S-H, C-N and N-H peaks of Cp have diminished while the peak at 1383 cm^−1^ in St-P(CL-b-EO)-AgNPs (typical for AgNP) gets minimized. The shifting and diminishing of characteristic peaks of drug and the minimization of AgNPs peak indicate a strong affinity of Cp with AgNPs. Furthermore, size of the St-P(CL-b-EO)-AgNPs has considerably decreased while their stability increased after its complexation with Cp, as shown by zeta sizer and zeta potential studies [33].

### 3.4. Encapsulation efficiency of St-P(CL-b-EO)-silver nanoparticles for cephalexin

The amount of Cp encapsulated in St-P(CL-b-EO)-AgNPs was calculated by the difference between the total amount of Cp initially taken and free drug in the formulation after the complexation step, determined by UV spectrophotometry. The encapsulation efficiency of the formulation was found to be 65.26%.

### 3.5. Antibacterial analysis

#### 3.5.1. Growth inhibition efficiency against *Staphylococcus aureus* (ATCC-25923)

**Figure 7.**
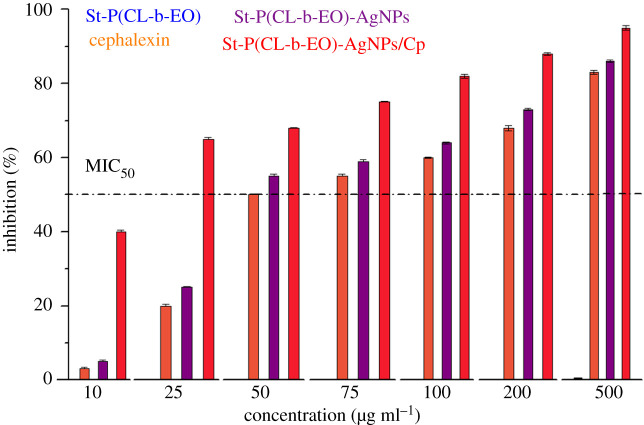
Growth inhibition efficiency as a function of concentration of St-P(CL-b-EO), cephalexin (Cp), St-P(CL-b-EO)-AgNPs and St-P(CL-b-EO)-AgNPs-Cp against *S. aureus*.

The growth inhibition efficiency of Cp, St-P(CL-b-EO), St-P(CL-b-EO)-AgNPs and St-P(CL-b-EO)-AgNPs/Cp were appraised against *S. aureus* through tetrazolium microplate assay. All the test samples were incubated against the selected microorganism in a concentration range of 10–500 µg ml^−1^ and their inhibition efficiency was evaluated. The results of inhibition efficiency as a function of concentration are summarized in figure 7. St-P(CL-b-EO) did not show any activity against *S. aureus* (ATCC-25923) with the highest tested concentration of 500 µg ml^−1^. Independent administration of Cp and St-P(CL-b-EO)-AgNPs were able to achieve 50% inhibition efficacy (MIC_50_) at 50 µg ml^−1^ concentration. The inhibition efficiency was dramatically increased by delivery of Cp by its complexation with St-P(CL-b-EO)-AgNPs [St-P(CL-b-EO)-AgNPs/Cp]; the application of only 25 µg ml^−1^ was able to inhibit the growth of *S. aureus* (ATCC-25923) to 65% (15% more than MIC_50_). This enhanced growth inhibition efficiency is attributed to the synergistic combination of Cp with St-P(CL-b-EO)-AgNPs through complexation that enhances its transport through the bacterial cell wall owing to their adherence to proteins and their affinity for sulfur in cellular metabolism. The numerical data are presented in the electronic supplementary material, table S1.

#### 3.5.2. Biofilm growth inhibition efficiency against *Staphylococcus aureus* (ATCC-25923)

**Figure 8.**
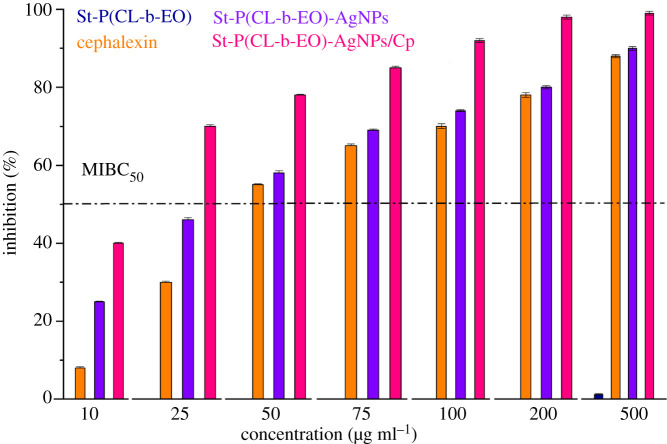
Biofilm growth inhibition efficiency as a function of concentration of St-P(CL-b-EO), cephalexin (Cp), St-P(CL-b-EO)-AgNPs and St-P(CL-b-EO)-AgNPs/Cp against *S. aureus*.

Biofilms are microbial populations embedded in a self-producing matrix that develop on living and nonliving solid surfaces. Biofilm inhibition is considered as a major drug target for the treatment of various microbial infections. An increased biofilm resistance to conventional treatments paves the way for the development of new strategies for efficient drug administration. The improvement in the antibacterial activity of Cp by delivery through St-P(CL-b-EO)-AgNPs/Cp is further endorsed by assay against biofilm growth inhibition efficiency, figure 8. St-P(CL-b-EO) did not show any antibiofilm activity against the target bacterial strain. Cp achieved 54% biofilm inhibition efficacy (MIBC_50_) at a concentration of 50 µg ml^−1^, whereas biofilm inhibition efficiency of 58% is achieved by St-P(CL-b-EO)-AgNPs at a concentration of 50 µg ml^−1^ (MIBC_50_). The biofilm inhibition efficiency of a complex of Cp with St-P(CL-b-EO)-AgNPs [St-P(CL-b-EO)-AgNPs/Cp] is enhanced several-fold, and more than 70% of inhibition was achieved while using only 25 µg ml^−1^. Enhanced antibiofilm activity of St-P(CL-b-EO)-AgNPs loaded Cp is attributed to the penetrative nature of the drug cargo St-P(CL-b-EO)-AgNPs through the bacterial cell wall. The numerical data are presented in the electronic supplementary material, table S2.

#### 3.5.3. Topographic imaging of *Staphylococcus aureus* colonies by atomic force microscopy

**Figure 9.**
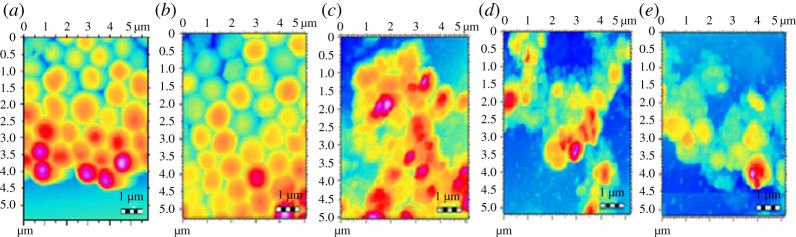
AFM topographic images of *S. aureus* bacterial cells: (*a*) control; after treatment at MIC_50_ with (*b*) St-P(CL-b-EO), (*c*) Cp, (*d*) St-P(CL-b-EO)-AgNPs, (*e*) St-P(CL-b-EO)-AgNPs-Cp.

**Figure 10.**
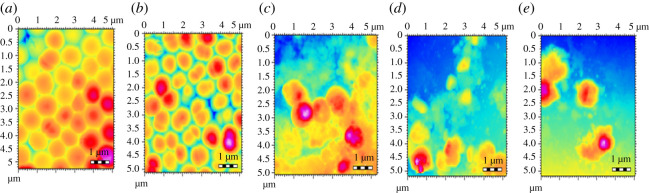
AFM topographic images of *S. aureus* bacterial cells biofilms: (*a*) control; after treatment at MIBC_50_ with (*b*) P(EO-b-CL), (*c*) Cp, (*d*) P(EO-b-CL)-AgNPs, (*e*) P(EO-b-CL)-AgNPs-Cp.

AFM was employed for visualization of the destruction of the bacterial cell walls and colonies of *S. aureus* by Cp, St-P(CL-b-EO), St-P(CL-b-EO)-AgNPs and St-P(CL-b-EO)-AgNPs/Cp at their MIC_50_ and MIBC_50_. The control cells of *S. aureus* have smooth, healthy and organized cell walls, figure 9*a*. St-P(CL-b-EO) did not cause any changes in the bacterial cell wall, thus confirming its inactivity towards the tested bacterial strain, figure 9*b*. The bacterial cells were denatured while treated with Cp at its MIC_50_ (50 µg ml^−1^), figure 9*c*. Similarly, St-P(CL-b-EO)-AgNPs at its MIC_50_ (50 µg ml^−1^) was able to achieve comparable destruction, figure 9*d*. The drug activity enhanced upon its loading in St-P(CL-b-EO)-AgNPs and complete destruction of the bacterial population is achieved at 25 µg ml^−1^ (MIC_50_). The bacterial population was converted into molten mass-like structures, figure 9*e*. The AFM analysis of biofilm inhibition efficiency has also endorsed the assay results as shown by topographic imaging of the biofilms after treatment with the drug and its conjugates at their respective MIBC_50_, figure 10.

## 4. Conclusion

In this study, St-P(CL-b-EO)-AgNPs were employed as an enhancer in the antibacterial activity of Cp through complexation. The synergistic effect of complexation of St-P(CL-b-EO)-AgNPs with Cp resulted in reduction in the MIC_50_ and MIBC_50_ by at least one order of magnitude. The visual destruction of bacterial cell walls and their biofilms was clearly observed by application of the drug and its conjugates at their respective MIC_50_ and MIBC_50_ through AFM topographic imaging. Hence, the co-formulation of antibiotics with NPs provides an excellent tool to counter the unresolved problem of increasing resistance of pathogenic bacteria against common antibiotics. Furthermore, the combination of antibiotic with NPs could be a realistic approach for the reduction in the amount of antibiotics use. The study indicates the high efficiency of drug encapsulation by polymer-stabilized AgNPs. Furthermore, the inherent possibility of polymers for tailoring their total molar mass, molar mass of individual segments, chemical composition and architecture, opens new horizons for further enhancement in drug efficacy.

## Supplementary Material

Figure S1

Reviewer comments

## Supplementary Material

Figure S2

## Supplementary Material

Table S1

## Supplementary Material

Table S2

## Data accessibility

Electronic supplementary material contains SEC elugrams, and NMR spectra of precursors and final product. The numerical data of the growth inhibition efficiency and biofilm growth film inhibition is also included.

## References

[1] Le Ouay B, Stellacci F 2015 Antibacterial activity of silver nanoparticles: a surface science insight. Nano Today 10, 339–354. (10.1016/j.nantod.2015.04.002)

[2] Sotiriou GA, Pratsinis SE 2010 Antibacterial activity of nanosilver ions and particles. Environ. Sci. Technol. 44, 5649–5654. (10.1021/es101072s) 20583805

[3] Kim S-H, Lee H-S, Ryu D-S, Choi S-J, Lee D-S 2011 Antibacterial activity of silver-nanoparticles against *Staphylococcus aureus* and *Escherichia coli*. Korean J. Microbiol. Biotechnol. 39, 77–85.

[4] Köck R et al. 2010 Methicillin-resistant *Staphylococcus aureus* (MRSA): burden of disease and control challenges in Europe. Eurosurveillance 15, 19688 (10.2807/ese.15.41.19688-en) 20961515

[5] Honda H, Krauss MJ, Jones JC, Olsen MA, Warren DK 2010 The value of infectious diseases consultation in *Staphylococcus aureus* bacteremia. Am. J. Med. 123, 631–637. (10.1016/j.amjmed.2010.01.015) 20493464 PMC3606273

[6] Archer NK, Mazaitis MJ, Costerton JW, Leid JG, Powers ME, Shirtliff ME 2011 *Staphylococcus aureus* biofilms. Virulence 2, 445–459. (10.4161/viru.2.5.17724) 21921685 PMC3322633

[7] Azam A, Ahmed AS, Oves M, Khan MS, Habib SS, Memic A 2012 Antimicrobial activity of metal oxide nanoparticles against Gram-positive and Gram-negative bacteria: a comparative study. Int. J. Nanomedicine 7, 6003 (10.2147/IJN.S35347) 23233805 PMC3519005

[8] Zhang Y, Liu X, Wang Y, Jiang P, Quek S 2016 Antibacterial activity and mechanism of cinnamon essential oil against *Escherichia coli* and *Staphylococcus aureus*. Food Control 59, 282–289. (10.1016/j.foodcont.2015.05.032)

[9] Mun S-H et al. 2013 Synergistic antibacterial effect of curcumin against methicillin-resistant *Staphylococcus aureus*. Phytomedicine 20, 714–718. (10.1016/j.phymed.2013.02.006) 23537748

[10] Goss CH, Muhlebach MS 2011 Review: *Staphylococcus aureus* and MRSA in cystic fibrosis. J. Cystic fibrosis 10, 298–306. (10.1016/j.jcf.2011.06.002) 21719362

[11] Parish LC, Jorizzo JL, Breton JJ, Hirman JW, Scangarella NE, Shawar RM, White SM, Team SS. 2006 Topical retapamulin ointment (1%, wt/wt) twice daily for 5 days versus oral cephalexin twice daily for 10 days in the treatment of secondarily infected dermatitis: results of a randomized controlled trial. J. Am. Acad. Dermatol. 55, 1003–1013. (10.1016/j.jaad.2006.08.058) 17097398

[12] Li W-R, Xie X-B, Shi Q-S, Duan S-S, Ouyang Y-S, Chen Y-B 2011 Antibacterial effect of silver nanoparticles on *Staphylococcus aureus*. Biometals 24, 135–141. (10.1007/s10534-010-9381-6) 20938718

[13] Chen Y, Zhang Y, Zhang H, Liu J, Song C 2013 Biofouling control of halloysite nanotubes-decorated polyethersulfone ultrafiltration membrane modified with chitosan-silver nanoparticles. Chem. Eng. J. 228, 12–20. (10.1016/j.cej.2013.05.015)

[14] Albanese A, Sykes EA, Chan WCW 2010 Rough around the edges: the inflammatory response of microglial cells to spiky nanoparticle. ACS Nano 4, 2490–2493. (10.1021/nn100776z) 20496953

[15] Albanese A, Tang PS, Chan WCW 2012 The effect of nanoparticle size, shape, and surface chemistry on biological systems. Annu. Rev. Biomed. Eng. 14, 1–16. (10.1146/annurev-bioeng-071811-150124) 22524388

[16] Duncan R, Vicent MJ 2013 Polymer therapeutics-prospects for 21st century: the end of the beginning. Adv. Drug Deliv. Rev. 65, 60–70. (10.1016/j.addr.2012.08.012) 22981753

[17] Delplace V, Couvreur P, Nicolas J 2014 Recent trends in the design of anticancer polymer prodrug nanocarriers. Polym. Chem. 5, 1529–1544. (10.1039/C3PY01384G)

[18] DJ, Twibanire AK, Grindley TB 2014 Polyester dendrimers: smart carriers for drug delivery. Polymers 6, 179–213. (10.3390/polym6010179)

[19] Oltra NS, Nair P, Discher DE 2014 From stealthy polymersomes and filomicelles to ‘self’ peptide-nanoparticles for cancer therapy. Ann. Rev. Chem. Biomol. Eng. 5, 281–299. (10.1146/annurev-chembioeng-060713-040447) 24910917 PMC4387849

[20] Martinho N, Damgé C, Reis C 2011 Recent advances in drug delivery systems. J. Biomater. Nanobiotechnol. 02, 510 (10.4236/jbnb.2011.225062)

[21] Jahangirian H, Lemraski EG, Webster TJ, Rafiee-Moghaddam R, Abdollahi Y 2017 A review of drug delivery systems based on nanotechnology and green chemistry: green nanomedicine. Int. J. Nanomed. 12, 2957–2978. (10.2147/IJN.S127683) PMC5396976 28442906

[22] Khalid S, Parveen S, Shah MR, Rahim S, Ahmed S, Malik MI 2019 Calixarene coated gold nanoparticles as a novel therapeutic agent. Arab. J. Chem. 13, 3988–3996. (10.1016/j.arabjc.2019.04.007)

[23] Maneerung T, Tokura S, Rujiravanit R 2008 Impregnation of silver nanoparticles into bacterial cellulose for antimicrobial wound dressing. Carbohydr. Polym. 72, 43–51. (10.1016/j.carbpol.2007.07.025)

[24] Das M, Shim KH, An SSA, Yi DK 2011 Review on gold nanoparticles and their applications. Toxicol. Environ. Health Sci. 3, 193–205. (10.1007/s13530-011-0109-y)

[25] Durán N, Durán M, de Jesus MB, Seabra AB, Fávaro WJ, Nakazato G 2016 Silver nanoparticles: a new view on mechanistic aspects on antimicrobial activity. Nanomed. Nanotechnol. Biol. Med. 12, 789–799. (10.1016/j.nano.2015.11.016) 26724539

[26] Jiang B, Guo Y, Kim J, Whitten AE, Wood K, Kani K, Rowan AE, Henzie J, Yamauchi Y 2018 Mesoporous metallic iridium nanosheets. J. Am. Chem. Soc. 140, 12 434–12 441. (10.1021/jacs.8b05206) 30129750

[27] Li C, Iqbal M, Lin J, Luo X, Jiang B, Malgras V, Wu KCW, Kim J, Yamauchi Y 2018 Electrochemical deposition: an advanced approach for templated synthesis of nanoporous metal architectures. Acc. Chem. Res. 51, 1764–1773. (10.1021/acs.accounts.8b00119) 29984987

[28] Li C, Iqbal M, Jiang B, Wang Z, Kim J, Nanjundan AK, Whitten AE, Wood K, Yamauchi Y 2019 Pore-tuning to boost the electrocatalytic activity of polymeric micelle-templated mesoporous Pd nanoparticles. Chem. Sci. 10, 4054–4061. (10.1039/C8SC03911A) 31015946 PMC6457336

[29] Li C et al. 2018 Emerging Pt-based electrocatalysts with highly open nanoarchitectures for boosting oxygen reduction reaction. Nano Today 21, 91–105. (10.1016/j.nantod.2018.06.005)

[30] Balaji Raja R, Singh P 2012 Synergistic effect of silver nanoparticles with the cephalexin antibiotic against the test strains. Biores. Bull. 2, 171–179.

[31] Burdușel A-C, Gherasim O, Grumezescu A, Mogoantă L, Ficai A, Andronescu E 2018 Biomedical applications of silver nanoparticles: an up-to-date overview. Nanomaterials 8, 681 (10.3390/nano8090681) PMC6163202 30200373

[32] Brown AN, Smith K, Samuels TA, Lu J, Obare SO, Scott ME 2012 Nanoparticles functionalized with ampicillin destroy multiple-antibiotic-resistant isolates of *Pseudomonas aeruginosa* and *Enterobacter aerogenes* and methicillin-resistant *Staphylococcus aureus*. Appl. Environ. Microbiol. 78, 2768–2774. (10.1128/AEM.06513-11) 22286985 PMC3318834

[33] Rahim S, Bhayo AM, Shah MR, Malik MI 2019 Star-shaped poly (ethylene oxide)-block-poly (caprolactone) conjugated silver nanoparticles: a colorimetric probe for cephalexin in environmental, biological and pharmaceutical samples. Microchem. J. 149, 104048 (10.1016/j.microc.2019.104048)

[34] Woodruff MA, Hutmacher DW 2010 The return of a forgotten polymer—polycaprolactone in the 21st century. Prog. Polym. Sci. 35, 1217–1256. (10.1016/j.progpolymsci.2010.04.002)

[35] Harris JM 1992 Poly(ethylene glycol) chemistry. Berlin, Germany: Springer US.

[36] Kareem F, Bhayo AM, Imran M, Shah MR, Khan KM, Malik MI 2019 Enhanced therapeutic efficacy of clotrimazole by delivery through poly(ethylene oxide)-block-poly(*ε*-caprolactone) copolymer-based micelles. J. Appl. Polym. Sci. 136, 47769 (10.1002/app.47769)

[37] Irfan M, Bhayo AM, Musharraf SG, Malik MI 2018 Chromatographic characterization of amphiphilic di- and tri-block copolymers of poly(ethylene oxide) and poly(ε-caprolactone). J. Sep. Sci. 41, 3352–3359. (10.1002/jssc.201800465) 30010243

[38] Bhayo AM, Abdul-Karim R, Musharraf SG, Malik MI 2018 Synthesis and characterization of 4-arm star-shaped amphiphilic block copolymers consisting of poly(ethylene oxide) and poly(ε-caprolactone). RSC Adv. 8, 28 569–28 580. (10.1039/C8RA05000G) PMC9084348 35542455

[39] Piaru SP, Mahmud R, Perumal S 2012 Determination of antibacterial activity of essential oil of *Myristica fragrans* Houtt. using tetrazolium microplate assay and its cytotoxic activity against vero cell line. Int. J. Pharmacol. 8, 572–576. (10.3923/ijp.2012.572.576)

[40] Wherry EJ et al. 2007 Molecular signature of CD8+ T cell exhaustion during chronic viral infection. Immunity 27, 670–684. (10.1016/j.immuni.2007.09.006) 17950003

[41] Bae CH, Nam SH, Park SM 2002 Formation of silver nanoparticles by laser ablation of a silver target in NaCl solution. Appl. Surf. Sci. 197, 628–634.

[42] Rahim S, Ali SA, Ahmed F, Imran M, Shah MR, Malik MI 2017 Evaluation of morphology, aggregation pattern and size-dependent drug-loading efficiency of gold nanoparticles stabilised with poly (2-vinyl pyridine). J. Nanopart. Res. 19, 259 (10.1007/s11051-017-3933-4)

[43] Rahim S, Khalid S, Bhanger MI, Shah MR, Malik MI 2018 Polystyrene-block-poly(2-vinylpyridine)-conjugated silver nanoparticles as colorimetric sensor for quantitative determination of Cartap in aqueous media and blood plasma. Sens. Actuators B 259, 878–887. (10.1016/j.snb.2017.12.138)

