# Supplementary material for: Enhancement in the antibacterial activity of cephalexin by its delivery through star-shaped poly(ε-caprolactone)-block-poly(ethylene oxide) coated silver nanoparticles
Source: R Soc Open Sci. 2020 Oct 7;7(10):201097. doi: 10.1098/rsos.201097 (PMC7657908; doi:10.1098/rsos.201097)
Supplement: Table S1 [file rsos201097supp3.docx]

Table S1. Growth inhibition efficiency as a function of concentration of St-P(CL-b-EO), Cephalexin (Cp), St-P(CL-b-EO)-AgNPs, and St-P(CL-b-EO)-AgNPs-Cp against S. aureus

| **Concentration µg/ml** | **Growth Inhibition efficiency (%) against Staphylococcus aureus** | | | |
| --- | --- | --- | --- | --- |
|  | St-P(CL-b-EO) | Cephalexin (Cp) | St-P(CL-b-EO)-AgNPs | St-P(CL-b-EO)-AgNPs/Cp |
| 10 | - | 3 ±0.5% | 5±0.3% | 40 ±0.4% |
| 25 | - | 20 ±0.4% | 25 ±0.2% | 65 ±0.4% |
| 50 | - | 50 ±0.3% | 55 ±0.5% | 68 ±0.2% |
| 75 | - | 55 ±0.5% | 59 ±0.4% | 75 ±0.3% |
| 100 | - | 60 ±0.2% | 64 ±0.3% | 82 ±0.5% |
| 200 | - | 68 ±0.7% | 73 ±0.2% | 88 ±0.4% |
| 500 | 0.5 ± 0.1% | 83 ±0.5% | 86 ±0.4% | 95 ±0.6% |
