# Supplementary material for: Enhancement in the antibacterial activity of cephalexin by its delivery through star-shaped poly(ε-caprolactone)-block-poly(ethylene oxide) coated silver nanoparticles
Source: R Soc Open Sci. 2020 Oct 7;7(10):201097. doi: 10.1098/rsos.201097 (PMC7657908; doi:10.1098/rsos.201097)
Supplement: Table S2 [file rsos201097supp4.docx]

Table S2. Biofilm growth inhibition efficiency as a function of concentration of St-P(CL-b-EO), Cephalexin (Cp), St-P(CL-b-EO)-AgNPs, and St-P(CL-b-EO)-AgNPs/Cp against S. aureus

| **Concentration µg/ml** | **Biofilm Growth Inhibition efficiency (%) against Staphylococcus aureus** | | | |
| --- | --- | --- | --- | --- |
|  | St-P(CL-b-EO) | Cephalexin (Cp) | St-P(CL-b-EO)-AgNPs | St-P(CL-b-EO)-AgNPs/Cp |
| 10 | - | 08±0.3% | 25 ±0.2% | 40 ±0.2% |
| 25 | - | 30 ±0.2% | 46 ±0.5% | 70 ±0.4% |
| 50 | - | 55 ±0.3% | 58 ±0.6% | 78 ±0.2% |
| 75 | - | 65 ±0.5% | 69 ±0.3% | 85 ±0.4% |
| 100 | - | 70 ±0.6% | 74 ±0.2% | 92 ±0.5% |
| 200 | - | 78 ±0.6% | 80 ±0.4% | 98 ±0.5% |
| 500 | 1.2 ±0.2% | 88 ±0.4% | 90 ±0.5% | 99 ±0.5% |
